# Fully 3D-Printed Dry EEG Electrodes

**DOI:** 10.3390/s23115175

**Published:** 2023-05-29

**Authors:** Adele Tong, Praneeth Perera, Zhanna Sarsenbayeva, Alistair McEwan, Anjula C. De Silva, Anusha Withana

**Affiliations:** 1 School of Computer Science, The University of Sydney, Sydney, NSW 2006, Australia; aton6105@uni.sydney.edu.au (A.T.);; 2 Department of Electronic and Telecommunication Engineering, University of Moratuwa, Moratuwa 10400, Sri Lanka; 3 School of Biomedical Engineering, The University of Sydney, Sydney, NSW 2006, Australia; 4 Sydney Nano, The University of Sydney, Sydney, NSW 2006, Australia

**Keywords:** EEG, 3D printing, dry electrodes, conductive filament

## Abstract

Electroencephalography (EEG) is used to detect brain activity by recording electrical signals across various points on the scalp. Recent technological advancement has allowed brain signals to be monitored continuously through the long-term usage of EEG wearables. However, current EEG electrodes are not able to cater to different anatomical features, lifestyles, and personal preferences, suggesting the need for customisable electrodes. Despite previous efforts to create customisable EEG electrodes through 3D printing, additional processing after printing is often needed to achieve the required electrical properties. Although fabricating EEG electrodes entirely through 3D printing with a conductive material would eliminate the need for further processing, fully 3D-printed EEG electrodes have not been seen in previous studies. In this study, we investigate the feasibility of using a low-cost setup and a conductive filament, Multi3D Electrifi, to 3D print EEG electrodes. Our results show that the contact impedance between the printed electrodes and an artificial phantom scalp is under 550 Ω, with phase change of smaller than −30${}^{\circ }$, for all design configurations for frequencies ranging from 20 Hz to 10 kHz. In addition, the difference in contact impedance between electrodes with different numbers of pins is under 200 Ω for all test frequencies. Through a preliminary functional test that monitored the alpha signals (7–13 Hz) of a participant in eye-open and eye-closed states, we show that alpha activity can be identified using the printed electrodes. This work demonstrates that fully 3D-printed electrodes have the capability of acquiring relatively high-quality EEG signals.

## 1. Introduction

Electroencephalography (EEG) is used to monitor the changes in brain electrical activity by measuring the potential difference across different nodes of the scalp. Typically, EEG signals have amplitudes of around 10 μV to 100 μV [1]. Spontaneous EEG frequencies often range from 0.1 Hz to 100 Hz [2], while signals induced by the brain’s response to stimuli, known as event-related potentials (ERP), can reach the range of 1000 Hz to 3000 Hz [3,4]. However, due to the skull and the skin’s composition, the acquisition of EEG can be challenging. Contact impedance, contact noise, and external interference are amongst the most challenging aspects of EEG, and even high-quality equipment cannot guarantee good-quality readings.

Traditionally, EEG is performed in laboratory settings, where electrodes are placed onto the head by clinicians using conductive adhesives. Recent advancements in technology have allowed EEG signals to be monitored using wearable devices. This enables EEG acquisition to be performed without having the user confined to laboratories. It is important that the devices are able to address the needs and preferences of the user in order to maximise reading quality and promote user adherence. Dry electrodes, which are designed with pin-like or finger-like structures, are often preferred in wearable EEG devices due to their ability to address portability and usability requirements. Various forms of dry electrodes have been developed to improve comfort and signal quality with different hair types [5]. However, user needs are subjective in nature, and they differ greatly for each individual. For instance, everyone has a different perception of comfort and preference for appearance. To adapt to different user needs, it is highly desirable to incorporate customisability into the electrode design [5].

Previous studies have shown that 3D printing using non-conductive material is able to fabricate customised electrode shapes [6,7]. These non-conductive electrodes are often coated with a conductive material, such as silver/silver chloride (Ag/AgCl) inks, to allow brain signals to be acquired. While this method has achieved good results with low contact impedance and low noise, the added complexity of fabrication has counteracted the intuitive and straightforward nature of 3D printing. The method also compromises the reproducibility of the customised electrode shapes, as consistent shapes are hard to achieve during the Ag/AgCl ink coating and curing process [8]. In this study, we explore the feasibility of manufacturing EEG electrodes entirely through 3D printing using a conductive filament—Multi3D Electrifi [9].

To evaluate the performance of the fully 3D-printed electrodes, we used a method of characterisation of contact impedance. Similar to prior research [6,10], we used a gelatine phantom scalp as the substrate to collect EEG signals. Our results show that the printed electrodes are able to achieve low contact impedance that is comparable to commercially available dry electrodes. We further performed functional testing, where we collected EEG signals from a participant while the participant’s eyes were opened and then closed. Our findings demonstrate that the printed electrodes were able to collect signals with identifiable alpha activities.

Our paper is organised as follows. Section 2 describes the design and prototyping process of the printed electrodes, as well as the setup used to measure the contact impedance and to perform the functional test. Section 3 presents the results of the tests. These results are then discussed in Section 4, where potential limitations and future directions are also evaluated.

## 2. Materials and Methods

### 2.1. Electrode Design

**Figure 1:**
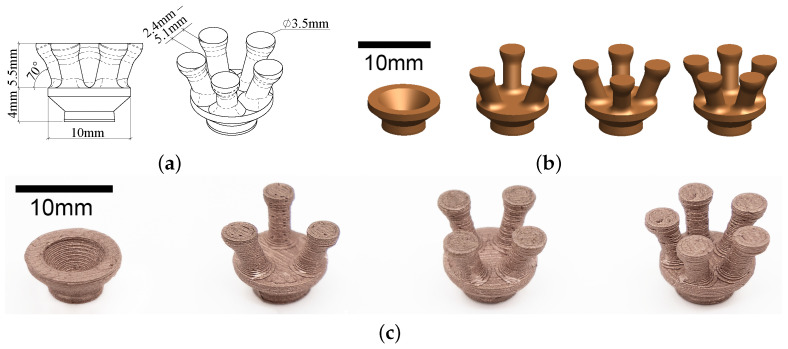
(**a**) Dimension of the 3D-printed electrode design; (**b**) Configurations with different number of pins (0 pin, 3 pins, 4 pins and 5 pins); (**c**) All configurations 3D-printed with Electrifi.

We designed the printed electrodes with reference to existing dry electrode designs [5,6,11]. We adopted rounded finger-like structures to help penetrate through hair, while reducing discomfort caused by traditional sharp, pin-like structures. We also took into account considerations relating to the limitations of the printer and filament. This includes avoiding overhang designs for better print quality, ensuring the design did not have details smaller than the chosen printer’s resolution of 0.2 mm, and avoiding structures that would fail easily with the brittleness of the material. Figure 1a presents the general dimension of the final electrode design.

Since one of the biggest advantages of 3D printing EEG electrodes is the ease of adjusting shapes and sizes to suit each individual’s anatomical features, it is important to look at how changing different parameters of a design would alter its performance. Previous publications have presented the effects of changing the number of pins on 3D-printed dry EEG electrodes [6,7]. We adopted a similar approach in the design of different electrode configurations. We present a 3-pin, a 4-pin, and a 5-pin design to investigate the effects of increasing the number of pins using the proposed fabrication approach. Instead of using 5–11 pins like previous studies reported [6,7], we minimised the number of pins to reduce the overall size of the electrode. A commercially available dry electrode—Neurospec dry Ag/AgCl electrode with 2 mm pins—was used to compare the results to the 3 pinned electrode designs. In addition, a flat, disc-shaped configuration with no pins (0-pin-flat) was designed with a similar diameter, shape, and contact area to the Neurospec disc-shaped dry Ag/AgCl electrode. The purpose of the 0-pin-flat configuration is to allow a comparison to be made between the printed electrode and the commercial electrode when the shape and size are identical. The 0-pin-flat configuration is expected to have a lower contact impedance than the ones with pins, as it has previously been found that a larger contact area reduces contact impedance [12]. All 4 design configurations are shown in Figure 1b, with the contact area, volume, and cost of material of each shown in Table 1.

### 2.2. Prototyping of Electrodes

**Figure 2:**
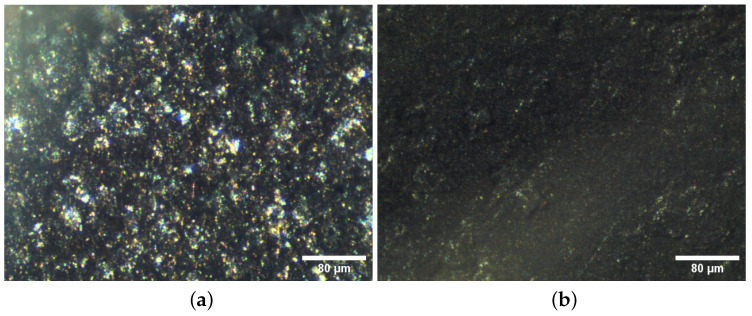
(**a**) Optical microscope image of an electrode printed with Electrifi; (**b**) Optical microscope image of a Neurospec dry Ag/AgCl electrode.

We used a copper-based filament, Electrifi, to print the EEG electrode designs. Scanning electron microscope (SEM) images and X-ray photoelectron spectroscopy (XPS) analysis conducted by previous studies indicate that the filament is mainly composed of thermoplastics and copper particles, with small traces of silver and other metals to reduce the rate of oxidation [13,14]. We present optical microscope images of an electrode printed with Electrifi and a Neurospec Ag/AgCl electrode in Figure 2 to show the surface morphology of the two types of electrodes. The image of the Electrifi electrode shows metallic flakes protruding out of the base material. This corresponds to previously presented SEM images [13,14]. While metallic particles are also visible in the image of the Neurospec Ag/AgCl electrode, these particles are smaller in size and are more dispersed than the ones present in the Electrifi electrode. To gain a deeper understanding of the composition of the conductive Electrifi filament, X-ray diffraction analysis (XRD) may be conducted in future studies.

The filament has previously been used for microstrip antennas, microchip transmission lines, and other electrical components due to its low resistivity [15,16,17]. Compared to other commercially available conductive filaments (e.g., Palmiga PI-ETPU, Black Magic 3D, and Proto-pasta), the resistivity of Electrifi is significantly lower, as shown in Table 2. Prior research has suggested the filament could be utilised in biomedical applications [18,19]. However, the application of the conductive filament in biological sensors has been rare. To date, Electrifi has not been reported for use in EEG applications.

We created 3D models of the electrode designs with computer-aided design (CAD) software, Solidworks, and exported them as Standard Template Library (STL) files to a slicing software, Simplify3D. We further adjusted printer settings using Simplify3D, which then generated G-code files for 3D printing. We used a low-cost 3D printer—The MakerGear M3—to produce the prints in this study. We applied a sheet of polyimide tape to the bed of the 3D printer to improve bed adhesion and ease of print removal. The printing temperature was set at the manufacturer-recommended temperature of 140 ${}^{\circ }$C [16]. This was significantly lower than conventional filaments to avoid conductivity deterioration due to excessive exposure to heat. Bed heating was also switched off to preserve conductivity. To compensate for the lack of bed heating, the first layer settings were adjusted to improve bed adherence. Layer height, print speed, and flow rate were adjusted to ensure the printed filament was as closely packed as possible. The optimal parameters we found after several trials are presented in Table 3. The electrodes we printed using these parameters are shown in Figure 1c.

### 2.3. Contact Impedance Testing

Impedance is the opposition of an alternating current (AC) and possesses both magnitude and phase [20]. Impedance is a combination of an imaginary component, reactance, and a real number component, resistance, and thus, is represented as a complex number [20]. The reactance is the difference between inductance ($X_{L}$) and conductance ($X_{C}$) and is a frequency-dependent component. Since EEG consists of AC signals at different frequencies, in this work, we measured impedance instead of resistance. Equation (1) shows the relationship between impedance (*Z*), resistance (*R*) and reactance (*X*). Impedance is often expressed in impedance magnitude and impedance phase to show the effects of both the resistance and the reactance.

(1) $$Z=\sqrt{R^{2}+{\left(X_{L}-X_{C}\right)}^{2}}$$

**Figure 3:**
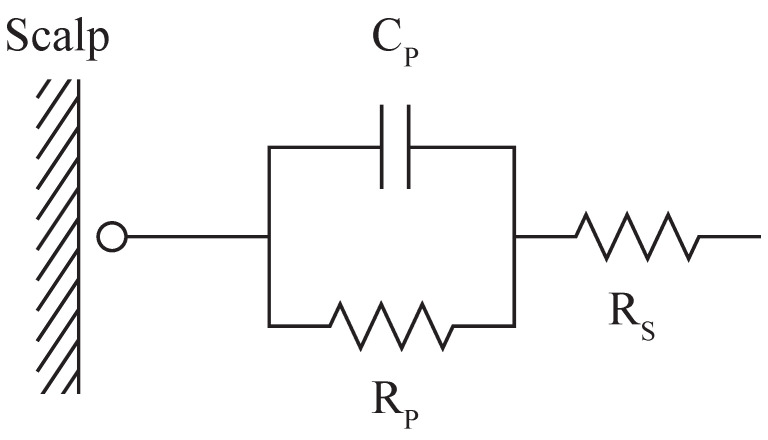
Electrical circuit demonstrating the contact impedance of a dry electrode to the skin, as adopted from Velcescu et al. [6] and Krachunov & Casson [7].

In the context of EEG, contact impedance between the scalp and the electrode plays a significant role in signal quality. Figure 3 represents the simplified electrical circuit of the electrode’s interface with the skin, where the series resistance ($R_{S}$) determines the resistance of the electrode material and the series component of the contact impedance; the parallel resistance ($R_{P}$) and parallel capacitance ($C_{P}$) determine the parallel component of the contact impedance between the skin and the electrode [6,7]. A reduction in contact impedance between the electrode and the scalp has been of interest in EEG recordings since high contact impedance could increase the noise level in recorded signals [20]. While contact impedance is not the sole electrical property that determines the quality of EEG signals, it is one of the biggest challenges dry electrodes face, and so is used as the characterisation method in many studies [6,7,10]. We follow the same approach.

**Figure 4:**
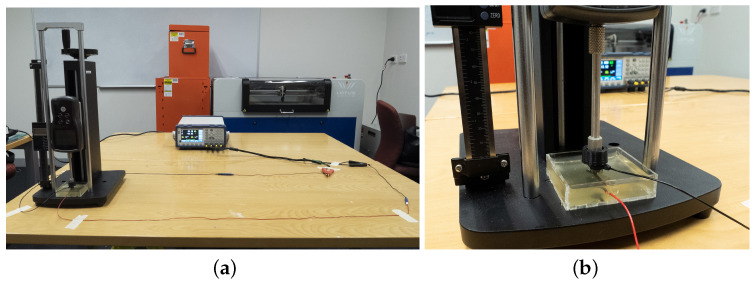
Setup for impedance testing: (**a**) The general setup showing the connection of the electrodes to the gelatine and the LCR meter; (**b**) The gelatine phantom scalp with embedded electrode.

Following similar studies in the literature [6,7,10], we used a phantom scalp with a conventional Ag/AgCl electrode embedded as the medium to measure the contact impedance of the printed electrodes. The phantom scalp was made of 10% gelatine and 90% saline solution, where the saline solution was prepared by mixing 1% sodium chloride (NaCl) with hot water. We used a cuboid of 5 cm × 5 cm × 1 cm of gelatine mixture to ensure the contact surface would remain even throughout all tests. We also used an LCR meter (BK Precision BK891) for measuring impedance. We connected the embedded electrode and the test electrode to either terminal of the LCR meter via standard DIN connectors. We applied a constant perpendicular force of 0.5 N to the electrode using a force gauge in order to maintain consistent contact between the gelatine and all electrode pins. We covered the tip of the force gauge with a 3D-printed polylactic acid (PLA) cap to prevent direct contact with the electrode. Cables were arranged and taped to the table to avoid interference. Figure 4 shows the setup of the experiment. To minimise the effects of filament oxidation and gelatine substrate deterioration, we measured all readings in one day using electrodes printed within the same day. In addition, 3 measurements were taken for each electrode in random order.

Previous studies report a commonly used frequency range of 1 Hz to 10 kHz when characterising contact impedance of EEG electrodes [6,7]. Due to the limitation of the model of the LCR meter used in this study, we could not test frequencies under 20 Hz. Instead, we selected the frequency range of 20 Hz to 10 kHz.

### 2.4. Functional Testing

We conducted a functional test to verify the functional performance of the printed electrodes. We selected the eye-open, eye-closed test for this purpose since the test is common amongst existing studies introducing new EEG electrode designs and fabrication processes [6,7,10,11,21]. Elaborate eye-open, eye-closed tests are used in clinical research studies to record resting state EEG rhythms [22]. The functional test included observing the collected EEG signals when a participant opens or closes their eyes. Due to an effect known as the Berger effect, the alpha band frequency (7–13 Hz) tends to increase when the eyes are closed, causing the amplitude of alpha signals to be amplified. In our test, we collected EEG signals when a participant opens or closes their eyes for a short duration of time. We aimed to evaluate the performance of the printed electrodes by evaluating their ability to record alpha activity.

The participant we recruited was a 24-year-old male with short hair (approximately 5 cm long). We used the OpenBCI Cyton board to collect EEG data, sampled at 250 Hz. We used the OpenBCI GUI to monitor the data in real time. During the procedure, we placed the test electrodes on the FP1, FP2, O1, and O2 nodes of a participant’s scalp, according to the 10–20 EEG system [23]. We placed a ground and a reference point on the scalp of the participant. Unipolar electrodes were used in the test to compare each channel with a common referential electrode. Placement of all electrodes is shown in diagrams presented in Section 3. We secured these electrodes on the participant’s head using a store-bought headband. The participant was then instructed to keep their eyes open for 5 s and then closed for 5 s. We performed a hand gesture to notify the participant to close their eyes. The participant was seated and asked to keep still during the entire data collection process. During the test, we used a configuration with 5 pins for the printed electrode since it performed better than the 4-pin configuration in the impedance test. Moreover, we found that it was more stable sitting on the scalp compared to the 3-pin configuration. We used dry electrodes from Neurospec as a comparison of performance against the proposed Electrifi-printed electrodes.

After recording the data, we applied a notch filter of 50 Hz to eliminate power line noise. To analyse alpha modulation, we visualised data related to eyes opened and eyes closed in both time and frequency domains separately. A band-pass filter of 7–13 Hz was applied to the time domain plot to show the acquired alpha-band frequencies. The frequency domain plot was the power spectral density estimation based on Welch’s method.

The test was performed in accordance with the rules of the Declaration of Helsinki of 1975 (revised in 2013). Ethics approval was given by the University of Sydney Human Research Ethics Committee (HREC) on 12 August 2019 (Application Number 2019/553). Informed consent was obtained from the participant involved in the study.

## 3. Results

### 3.1. Contact Impedance Measurement

Using the measured impedance measurements at various frequencies, we calculated the $R_{S}$, $R_{P}$, and $C_{P}$ components illustrated in Figure 3. We present these values for each configuration in Table 4.

**Figure 5:**
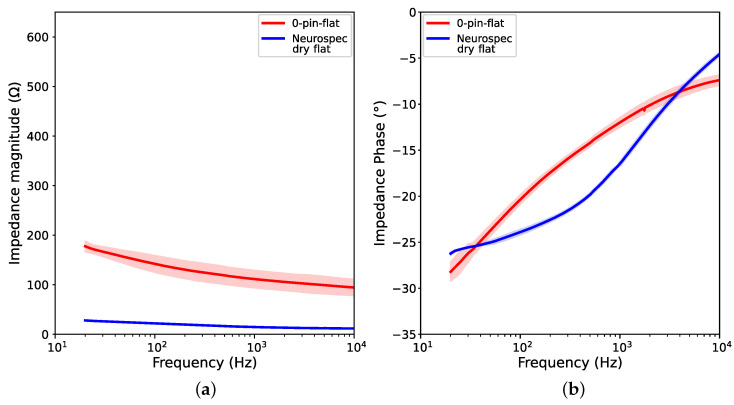
Graphs showing impedance measurements of printed 0-pin-flat electrode and Neurospec flat electrode at different frequencies: (**a**) Impedance magnitude; (**b**) Impedance phase.

**Figure 6:**
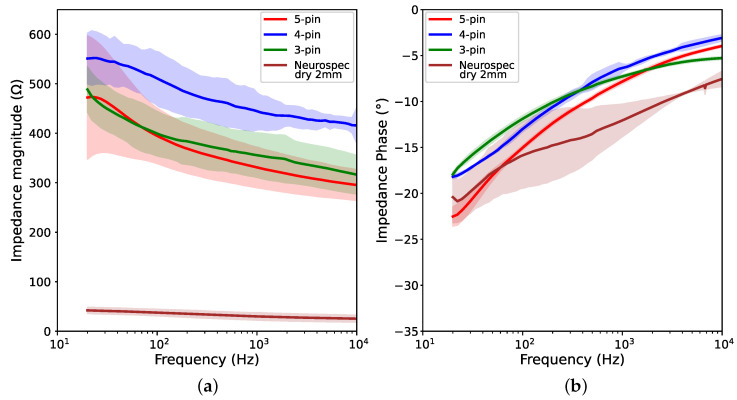
Graphs showing impedance measurements of 3-pin, 4-pin, and 5-pin electrodes and Neurospec dry 2 mm electrode at different frequencies: (**a**) Impedance magnitude; (**b**) Impedance phase.

We present the results of contact impedance measurements, including both the impedance magnitude and phase in Figure 5 and Figure 6. The values from the three trials of each electrode are plotted into semi-logarithmic graphs with a confidence interval of 95%. First, we compared the performance of the printed 0-pin-flat electrode to the Neurospec flat electrode, to demonstrate the performance of Electrifi when the contact area is similar to the commercially available electrode. Then, we present the results of electrodes with different numbers of pins.

#### 3.1.1. Results for 0-Pin-Flat Electrode

Figure 5 shows that the 0-pin-flat electrode has low impedance measurements throughout the entire range of test frequencies, with a maximum of 180 Ω at 20 Hz and a minimum of 100 Ω at 10 kHz. The printed 0-pin-flat electrode shows a very slow and stable increase as frequency decreases. The phase angle at 20 Hz is approximately −28${}^{\circ }$ for the 0-pin-flat electrode, and −26${}^{\circ }$ for the Neurospec flat electrode. Although the 0-pin-flat electrode performs worse than the commercial dry electrode, which has contact impedance between 10 and 30 Ω, the results of the 0-pin-flat electrode are comparable to findings reported previously [6]. In the prior work, the best-performing electrode, fabricated by 3D printing and Ag/AgCl coating, reported a contact impedance of approximately 450 Ω at 1 kHz and 550 Ω at 20 Hz [6]. Despite having a smaller contact surface area, the 0-pin-flat electrode fabricated in this study has shown a lower magnitude of contact impedance at 20 Hz.

#### 3.1.2. Results for Different Numbers of Electrode Pins

Figure 6 shows that the changes in contact impedance throughout all test frequencies for the 3-pin, 4-pin, and 5-pin electrodes are in close ranges. The contact impedance measurement is between 325 and 500 Ω for the 3-pin electrode, and between 300 and 475 Ω for the 5-pin electrode. The 4-pin electrode shows a slightly higher contact impedance magnitude, ranging from approximately 425 to 550 Ω. For all 3 design configurations, the change of impedance magnitude is under 200 Ω, and the difference in magnitude between different configurations is under 150 Ω at all frequencies. These values are significantly greater than the Neurospec dry 2 mm electrode, which shows an impedance magnitude of 25–42 Ω, and a magnitude change of 17Ω. The phase angles for the 3-pin, 4-pin, and 5-pin electrodes at 20 Hz are approximately −18${}^{\circ }$, −18${}^{\circ }$, and −23${}^{\circ }$, respectively. These are similar to the Neurospec 2mm electrode, which has a phase angle of −21${}^{\circ }$ at 20 Hz. Previous works examining 3D-printed and Ag/AgCl coated EEG electrodes have reported contact impedance of 500–1000 Ω at 20 Hz for electrodes with different numbers of pins and an impedance phase of up to −50${}^{\circ }$ [6]. This suggests that the effect of decreasing the number of pins and the contact area is less significant in this study, as the difference in contact impedance between different configurations is low.

### 3.2. EEG Recording

**Figure 7:**
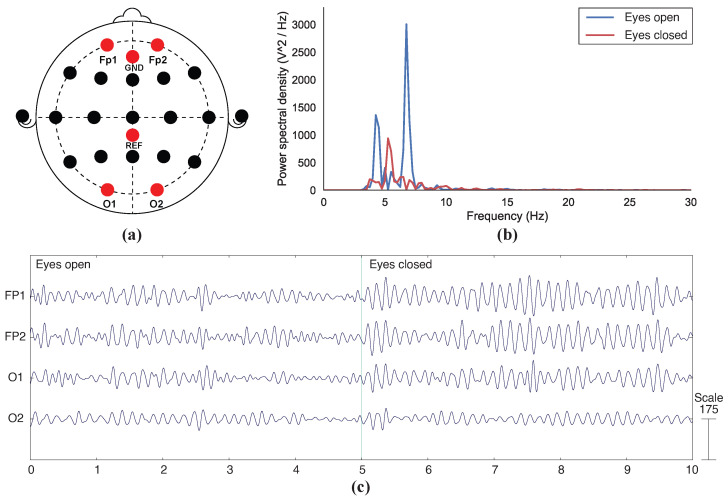
Functional test results for electrodes 3D-printed with Electrifi: (**a**) Location of recorded nodes according to the 10–20 system; (**b**) Power spectral density graph during eye-open and eye-closed states; (**c**) Electroencephalography (EEG) signals collected during a 5-s eye-open phase and a 5-s eye-closed phase.

**Figure 8:**
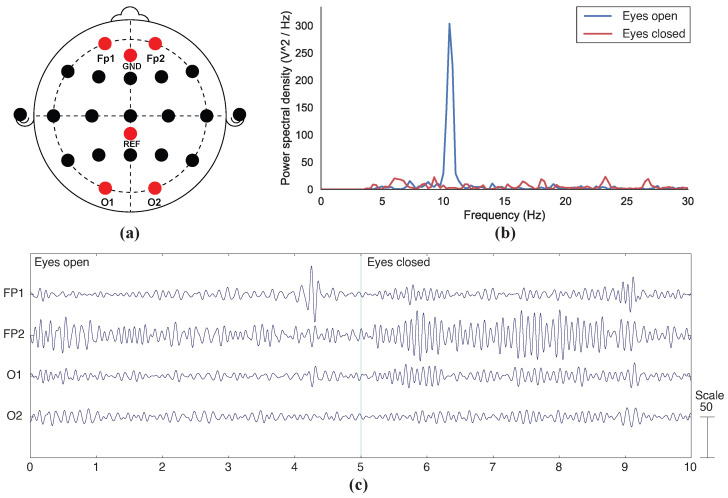
Functional test results for Neurospec’s commercially available dry electrodes: (**a**) Location of recorded nodes according to the 10–20 system; (**b**) Power spectral density graph during eye-open and eye-closed states; (**c**) EEG signals collected during a 5-s eye-open phase and a 5-s eye-closed phase.

Figure 7 and Figure 8 show that aroused alpha activities can be observed through EEG signals acquired with both 3D-printed and commercial electrodes when a participant opens and closes their eyes. In Figure 7c, the amplitude of the EEG oscillations shows a significant increase during the eye-closed state, especially for electrodes FP1, FP2, and O1, compared to that of the first 5 s, when the eyes were open. The changes in electrode O2 were less significant. In the power spectral density graph of Figure 7b, the peak value of approximately 7.5 Hz is found to be most dominant when the eyes were closed, whereas during the eye-open state, the peak is a lot less significant. A secondary peak can be seen at around 4.5 Hz during the eye-closed state.

The Neurospec dry electrodes also show a significant increase in alpha activities for FP1, FP2, and O1 during the eye-closed state (Figure 8c), while the difference is less obvious for O2. The power spectral density of Neurospec electrodes shows a significant peak at around 10.5 Hz when eyes were closed (Figure 8b), with no obvious peak observed in the eye-open state. In summary, the results show that the proposed 3D-printed electrodes can be used in the functional application of EEG sensing.

## 4. Discussion

### 4.1. Contact Impedance

The impedance measurements show promising results for electrodes printed with Electrifi, with readings of all configurations well under the 10 kΩ limit for passive electrodes throughout all test frequencies [5]. This suggests that the printed electrodes could be used to collect brain signals, without the need for active shielding, which is a noise reduction method generally used for contact impedance of up to 40 kΩ [5]. Without the use of heavy cables and components needed for active shielding, the overall size and bulkiness of the device could be reduced.

Although the contact impedance of the printed electrodes was higher than that of the commercial Neurospec dry electrodes, the results appeared to be significantly better than the reported 3D-printed electrodes with Ag/AgCl coating [6]. The 3D-printed and Ag/AgCl coated electrodes proposed by Casson and Mahdi [7] had contact impedance ranging from 2 kΩ to over 10 kΩ at 20 Hz, and those proposed by Velcescu et al. [6] had contact impedance of 500–3000 Ω at 20 Hz. The contact impedance ranges of 300–550 Ω achieved by the electrodes with pins fabricated in this study, and 100–180 Ω achieved by the 0-pin-flat electrode, are significantly lower in comparison. It is worth noting that the increase in impedance magnitude and phase are not consistent with the change in the number of pins. For instance, despite having a larger contact area, the 4-pin electrode had a higher impedance magnitude than the 3-pin electrode (Figure 6a). Additionally, the 5-pin electrode, which had the largest contact area in the 3 configurations, displayed a more similar impedance magnitude to the 3-pin electrode than the 4-pin electrode. Such inconsistencies can also be observed in studies involving 3D-printed electrodes with conductive coatings [6,7]. Nevertheless, differences in both impedance magnitude and phase across the three fully 3D-printed electrodes are notably less significant than those reported previously [6,7]. However, the setups used to conduct the tests were difficult to replicate. In particular, there are multiple factors that could affect the performance of the gelatine phantom scalp [24,25]. Thus, it is hard to conduct a direct comparison between studies. Further verification of improvement in contact impedance performance is needed to be able to compare electrodes fabricated using different methods.

Moreover, different configurations of printed electrodes have shown comparable results in contact impedance readings. Previous studies involving 3D-printed electrodes have shown a great effect of increasing the number of pins, with impedance magnitude drastically increasing while the number of pins is reduced [6]. In comparison, different configurations of fully 3D-printed electrodes in this work showed relatively small differences of under 150 Ω in contact impedance magnitude and phase change of smaller than −30${}^{\circ }$ across different configurations. One reason for this could be the difficulty in controlling the Ag/AgCl inks distribution in previous studies, whereas 3D printing is able to produce consistent results with the same printer settings. By fabricating electrodes entirely through 3D printing, the quality of electrodes can be controlled, leading to more consistent results. The results suggest that customisation of EEG electrodes is possible through 3D printing with Electrifi, without significantly impacting signal quality.

### 4.2. Functional Testing

The functional test results showed that changes in alpha waves can be detected with both 3D-printed and commercial electrodes (Figure 7b,c). The burst of alpha activities can be clearly observed during the last 5 s in Figure 7b and Figure 8b when the participant’s eyes were closed. The alpha-band EEG signals collected using the fully 3D-printed displayed a visually similar pattern to those presented from previous eye-open, eye-closed tests [11,26,27]. While changes in EEG signals were observed in all four channels for both types of electrodes (Figure 7c and Figure 8c), the FP1, FP2, and O1 nodes showed more prominent changes than that of O2 in both cases. Since this trend was consistent amongst both types of electrodes, it suggests that the difference in node readings could be caused by the test setup. For instance, inconsistent pressure applied across different channels is a likely factor to contribute to the inconsistent results between different nodes [23], as electrodes were attached to the head using a headband without adjustment to force that was applied to individual electrodes.

The peak frequencies for the 3D-printed and commercial electrodes when eyes were closed were approximately 7.5 Hz and 10.5 Hz, respectively, (Figure 7c and Figure 8c), which were both in the alpha band. For both types of electrodes, the peaks during the eye-closed state were notably more obvious as compared to the eye-open state. This indicates that the alpha waves were the most prominent signal during the eye-closed state. The pattern in power spectral density between eye-open and eye-closed states reported in this study is similar to that of the 3D-printed electrodes coated with conductive pastes presented in a previous study [10]. While an unexpected secondary peak was observed in the 3D-printed electrode plot (Figure 7b), a similar peak pattern can also be observed in the brush-type design from the same study [10]. Although it was assumed to be caused by noise, more extensive tests would need to be carried out to verify.

### 4.3. Limitations

#### 4.3.1. Customisation Challenges

Although this study shows that similar contact impedance could be achieved with different design configurations, the number of pins was the only variable being examined. In future work, it is necessary to investigate a greater variety of forms to verify the degree of customisability achievable using our proposed approach. Furthermore, our approach is not directly customisable by end users. The process of design and fabrication would need to be streamlined to allow end users to directly modify electrode designs according to their needs. For example, a technological enabler that allows users to modify and print designs should be developed in the future to facilitate fabrication of customised electrodes.

#### 4.3.2. Fabrication Challenges

We acknowledge that the low printing temperature required to preserve the resistivity of Electrifi may raise other printing problems, such as poor adhesion to the printer bed and between layers, which greatly affect the print quality. However, due to the novelty of the filament, information on printing parameters is scarce and occasionally contradicting. For instance, the reported optimal layer height and printing speed vary amongst existing literature [15,16,28,29]. Furthermore, due to the rarity of utilising Electrifi in delicate structures, existing guidelines are not optimised for printing delicate pin-like structures. As a result, certain parameters found to be crucial in this study, such as infill outline overlap and minimum infill length, are omitted in previous publications. High-quality prints, which are required to achieve high-quality EEG signals, could only be achieved after multiple trials. A more comprehensive guideline needs to be developed for end users to be involved in the fabrication process, to avoid compromising the signal quality of customised design. In addition to the challenges of printing with Electrifi, the design constraints for 3D printing, such as consideration of tolerance and overhangs, also limit the degree of design flexibility. This may act as a barrier to user customisation, as it could be challenging for the user to fabricate their desired shape and size.

#### 4.3.3. Material Properties

The soft but brittle nature of Electrifi has shown to be a limitation of the material. During the fabrication and testing process, several prototypes were damaged as a result of the force applied to the pins. This acts as a constraint to the electrode design, as wider pins are required than the usual dry EEG electrodes. Furthermore, we found that the base of the electrode, where the clip lead was attached, becomes deformed over repeated use. The connection eventually became loose as a result of the softness of the filament, which significantly affected the signal quality. This connection problem needs to be addressed to allow the electrode to be used for longer periods of time.

Previous studies on Electrifi suggested that the 3D printing filament is prone to thermal oxidation [16,25]. However, information on the filament’s rate of oxidation over time was not available. In this work, we did not investigate the rate of oxidation and the effect of oxidised electrodes. Hence, it could potentially be crucial to determining the suitability of long-term usage of the printed electrodes, as their contact impedance and biocompatibility could be affected. More in-depth studies will be required to examine the possible approaches to delay the oxidation process or remove the oxidised layers in the future. Furthermore, future work also needs to investigate the biocompatibility of the material to ensure it is safe for long-term use.

#### 4.3.4. Study Limitations

We limited the contact impedance test performed in this study by the reproducibility of the phantom scalp. We found that the condition of the gelatine was deteriorating over time due to the evaporation of water, which altered its capacitance. Although we conducted all measurements within a single day to reduce the effects of gelatine deterioration, the effects of gelatine deterioration could not be entirely mitigated. This poses a challenge when comparing results to existing studies.

While the eye-open, eye-closed test conducted in this study has previously been performed by many as proof of concept [6,7,10,11,21,23], standard methodologies and a gold standard are yet to be established. Although the International Federation of Clinical Neurophysiology (IFCN) published recommendations for a similar test [22], the protocol was designed to collect resting state EEG in clinical research using a single type of electrodes. Some specific instructions, such as the use of 128–256 electrodes, the condition assessment of the subject prior to the test, and the probing of the subject’s transition to drowsiness, are less relevant to the goal of comparing electrode performance. There are no existing guidelines outlining the standard protocol for using and comparing different electrode types, which is the main challenge identified in novel EEG electrode validating tests [5]. Moreover, brain signals are not possible to replicate. Signals collected with the slightest spatial distance and time difference would result in a difference in reading, and thus, cannot be compared directly [5]. As such, although our work shows the possibility of detecting changes in alpha activities with fully 3D-printed electrodes, further work involving the acquisition of a wider frequency range of spontaneous EEG and ERP on more subjects is required to establish the quality of the signal the fully 3D-printed electrodes are able to achieve.

## 5. Conclusions

Our work has proposed to fabricate dry EEG electrodes through 3D printing with a conductive filament, Multi3D Electrifi. This fabrication approach utilises low-cost setup and accessible materials and eliminates the need for further processing, such as coating non-conductive 3D prints with Ag/AgCl coatings. The study results show that the fully 3D-printed electrodes are able to achieve better performance than previous studies involving Ag/AgCl coated electrodes, with all recorded contact impedance under 500 Ω. In addition, comparable results could be achieved with different configurations of design, suggesting that it is possible to customise shapes without a great reduction in signal quality using the proposed method. The printed electrodes were also able to detect alpha activities when performing a preliminary functional test commonly used to validate EEG electrode designs—the eye-open and eye-closed test. The proposed methodology shows the feasibility of utilising a straightforward fabrication process and accessible materials for EEG electrode customisation. This has the potential to act as a tool for customisation to be implemented at the end user’s level, enabling them to create EEG wearable devices catering to their own needs and preferences.

## Figures and Tables

**Table 1:** Contact surface area of each electrode configuration.

Electrode Configuration	Contact Surface Area (mm^2^)	Volume (mm^3^)	Cost of Material (USD)
0-pin-flat	42.41	130.22	0.68
3-pin	28.86	314.98	1.66
4-pin	38.48	350.89	1.84
5-pin	48.11	386.75	2.03

**Table 2:** Resistivity of different commercially available conductive filaments [17,19].

Filament	Resistivity (Ωcm)
Palmiga PI-ETPU	30–700
Black Magic 3D	0.6
Proto-pasta	30
Multi3D Electrifi	0.006

**Table 3:** Printing parameters used to print EEG electrodes.

Printing Parameters	Value
Nozzle diameter	0.35 mm
Print temperature	140 ${}^{\circ }$C
Bed temperature	25 ${}^{\circ }$C
Flow rate	110%
Print speed	15.0 mm/s
Primary layer height	0.16 mm
Infill outline overlap	50%
Minimum infill length	0.00 mm
First layer height	125%
First layer speed	40%

**Table 4:** Calculated series resistance ($R_{S}$), parallel resistance ($R_{P}$), and parallel capacitance ($C_{P}$) values for all electrode configurations.

Electrode Configuration	$R_{S}$ (Ω)	$R_{P}$ (Ω)	$C_{P}$ (μF)
Neurospec dry flat	11.67	16.61	58.18
Neurospec dry 2 mm	25.43	17.85	39.80
0-pin-flat	94.35	90.28	15.67
3-pin	316.47	178.95	10.15
4-pin	415.93	154.57	6.51
5-pin	295.47	212.31	8.27

## Data Availability

Not applicable.
